# Surface modification of polyester fabric using plasma-dendrimer for robust immobilization of glucose oxidase enzyme

**DOI:** 10.1038/s41598-019-52087-8

**Published:** 2019-10-31

**Authors:** Mohammad Neaz Morshed, Nemeshwaree Behary, Nabil Bouazizi, Jinping Guan, Guoqiang Chen, Vincent Nierstrasz

**Affiliations:** 1 Textile Material Technology, Department of Textile Technology, The Swedish School of Textiles, Faculty of Textiles, Engineering and Business, University of Borås, SE-50190 Borås, Sweden; 2 Ecole Nationale Supérieure des Arts et Industries Textiles (ENSAIT), GEMTEX Laboratory, 2 allée Louise et Victor Champier BP 30329, 59056 Roubaix, France; 3 Université de Lille, Nord de France, F-59000 Lille, France; 4 College of Textile and Clothing Engineering, Soochow University, 215006 Suzhou, China

**Keywords:** Immobilized enzymes, Surface chemistry, Porous materials, Biomaterials

## Abstract

Robust immobilization of glucose oxidase (GOx) enzyme was achieved on poly(ethylene terephthalate) nonwoven fabric (PN) after integration of favourable surface functional groups through plasma treatments [atmospheric pressure-AP or cold remote plasma-CRP (N_2_ + O_2_)] and/or chemical grafting of hyperbranched dendrimers [poly-(ethylene glycol)-OH or poly-(amidoamine)]. Absorption, stability, catalytic behavior of immobilized enzymes and reusability of resultant fibrous bio-catalysts were comparatively studied. Full characterization of PN before and after respective modifications was carried out by various analytical, instrumental and arithmetic techniques. Results showed that modified polyester having amine terminal functional groups pledged better surface property providing up to 31% enzyme loading, and 81% active immobilized enzymes. The activity of the enzyme was measured in terms of interaction aptitude of GOx in a given time to produce hydrogen peroxide using colorimetric assay. The immobilized GOx retained 50% of its original activity after being reused six (06) times and exhibited improved stability compared with the free enzyme in relation to temperature. The reaction kinetics, loading efficiency, leaching, and reusability analysis of enzyme allowed drawing a parallel to the type of organic moiety integrated during GOx immobilization. In addition, resultant fibrous bio-catalysts showed substantial antibacterial activity against pathogenic bacteria strains (*Staphylococcus epidermidis* and *Escherichia coli*) in the presence of oxygen and glucose. These results are of great importance because they provide proof-of-concept for robust immobilization of enzymes on surface-modified fibrous polyester fabric for potential bio-industrial applications.

## Introduction

Biomolecules, especially enzymes are receiving remarkable attention to multidisciplinary researchers due to significant activity, selectivity, and specificity which is suitable to catalyze many intricate chemical reactions in an unfavorable experimental environment. However, poor operational stability, as well as inadequate reusability of enzymes, greatly limits their industrial applications^1^. Glucose-1-oxidase (GOx) (β-D-glucose: oxygen-1-oxidoreductase) is a naturally produced redox enzyme that catalyzes the oxidation of β-D-glucose into D-glucono-1,5-lactone or D-glucono-δ-lactone and hydrogen peroxide by using molecular O_2_ as an electron acceptor (see reaction 1–2)^2–4^.

$${\rm{\beta }} \mbox{-} {\rm{D}} \mbox{-} {\rm{Glucose}}+{\rm{Enzyme}} \mbox{-} {\rm{FAD}}\,\to \,{\rm{Enzyme}} \mbox{-} {{\rm{FADH}}}_{2}+{\rm{D}} \mbox{-} {\rm{glucono}} \mbox{-} 1,5 \mbox{-} {\rm{lactone}}$$

$${\rm{Enzyme}} \mbox{-} {{\rm{FADH}}}_{2}+{{\rm{O}}}_{2}\,\to \,{\rm{Enzyme}} \mbox{-} {\rm{FAD}}+{\rm{Hydrogen}}\,{\rm{peroxide}}$$

GOx is being extensively used in various fields; such as food processing-additive, dairy and the lacto-peroxidase system, bread-making, dry egg powder, antioxidant/preservative (oxygen scavenger), D-glucono-δ-lactone (gluconic acid) production, glucose sensor/assay and so on^4–9^. However, as a relatively expensive catalyst, in many instances, the use of free GOx enzymes in a continuous system is economically undesirable even with its potential reusability if recovered. Immobilization of GOx can provide the prospects for easy recovery. Immobilization also provides great control to the design of the reactor and result of the reactions^10–13^. Several immobilization methods (physical adsorption, covalent bonding, entrapment and crosslinking) have been introduced for robust immobilization of GOx aiming to maintain the structure and spatial orientation of enzyme with high diffusion of/from the active site to provide a reassuring place for the biological reaction^14–17^. A number of materials have been introduced as a potential entrant for GOx immobilization; such as, carbon felt^4^, gold nanostars^16^, cellulose nanocrystals^18^, carbon nanotubes^19,20^, nanofibers^21^ and oxide metallic nanoparticles, etc.^22,23^. Due to high strength, inexpensiveness, biocompatibility, as well as resistance to most acids, oxidizing agents, microorganisms and chemical reagents^24–26^, polyester fiber is an ideal material for immobilization of GOx considering the various bio/electrochemical application such as sensing, biosynthesis, catalysis etc.^27,28^.

However, poor immobilization and blocking of accessibility of active sites of the enzyme remain a contrary drawback of enzyme immobilization. The performance of immobilized enzymes is greatly influenced by the surface chemical feature of support material, i.e. functional groups present on the surface. The presence of favorable functional groups on the surface can boost the integration of enzyme ensuring high loading of active enzymes, and poor leaching during applications^29^. Since the polyester surface is highly inert, with low surface energy, a favorable surface chemical feature, with appropriate surface functional groups is necessary. Several surface modification method for polyester has been reported; such as photo-induced irradiation^30^, electron beam irradiation^31^, enzymatic modification^32^ and plasma treatments^33^. Among them, considering the cost-effectiveness and eco-friendliness (no harmful solvent, no chemical waste and less degradation of the specimen), the plasma treatment based surface modification is more desirable^29^. Several studied has reported the effectivenss of plasma treatment to introduce functional groups such as hydroxyl, carboxyl, amine, amide etc. by selecting the appropriateplasma-discharge parameters (i.e. type of gas, treatment time, input power etc.)^34,35^. The change in nano-roughness (~10 nm) has been shown to have a significant affect on the protein attachment^34^ when oxygen^25^ as well as nitrogen^36^ containing plasma has been used^37^. Therefore, the plasma-discharge parameters should be carefully selected to reach the desired modification that further ensures high surface energy, and therefore improves the biocompatibility without altering substance primary properties.

The main goal is to provide favorable surface features for robust enzyme immobilization. A selected terminal group based hyperbranched polymers (dendrimers) was also chosen to provide further favorable functional groups proving their eco-friendliness and ability to form complexes via molecular encapsulation, covalent and non-covalent interactions^38^. A number of studies already reported the effectiveness of hyperbranched dendrimers on the immobilization of various catalysts^39–44^. To the best of our knowledge, so far no report has introduced any such approach for robust immobilization of glucose oxidase on surface-modified polyester fabric by using plasma and dendrimers. This study report here for the first time, the influence of favorable functional groups introduced by surface modification of polyester using plasma surface treatment and hyperbranched dendrimers to achieve robust immobilization of enzyme. Air atmospheric plasma; and cold remote plasma with N_2_ + O_2_ has been used to activate the hydrophobic surface of polyester fabric and/or grafting of hyperbranched dendrimers with different terminal functional groups followed by immobilization of GOx. The effect of plasma treatment on PN has been studied in terms of wettability (water contact angle- ${{\rm{\theta }}}_{{{\rm{H}}}_{2}{\rm{O}}}$ and capillary uptake) and x-ray photoelectron spectroscopy (XPS) analysis. The report will further discuss the bio-catalytic activity of glucose oxidase (before and after immobilization) in terms of their ability to produce hydrogen peroxide through colorimetric assay. Qualitative analysis by means of scanning electron microscope analysis and quantitative analysis through diverse arithmetic methods has been chosen to investigate the GOx loading, immobilization, and stability.

## Experimental

### Chemicals

**Figure 1:**
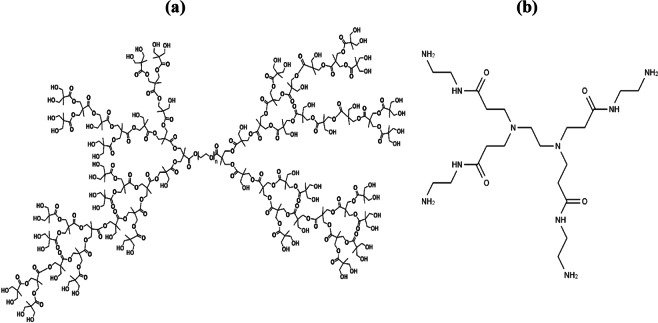
Chemical structure of (**a**) Hyperbranched poly-(ethylene glycol) Pseudo dendrimer having hydroxyl (–OH) end group^61^ and (**b**) poly-(amidoamine) dendrimer consists of ethylenediamine core and tertiary amine branches^62^.

Hyperbranched poly-(ethylene glycol)–OH (PEG–OH) dendrimer, poly-(amidoamine) (PAMAM) dendrimer (see Fig. 1), glucose oxidase (E.C. 1.1.3.4) enzyme, petroleum ether, ethanol (C_2_H_5_OH) and β-D-Glucose (C_6_H_12_O_6_) were purchased from Sigma-Aldrich Ltd. All purchased chemicals used in this study were of analytical grade and used as received. Deionized water from a water purification system provided by GFL-Gesellschaft für Labortechnik mbH (Germany) was used throughout all the experiments.

### Polyester fabrics

Poly-(ethylene terephthalate) fabric was fabricated in European nonwoven platform-CENT (France) based on a web of fibers (average diameter 12 µm) formed by carding and consolidated by hydro-entanglement (entangling with water jets). The characteristics of nonwoven are displayed in Table S1 of supplementary information. Surface impurities of fiber such as dust, spinning oil and contaminants were extracted from polyester fabric by using the Soxhlet extraction method (see Fig. S1) as described elsewhere^26,33^. The removal of impurities was assessed by the water break test where the surface tension of sample water (from PN rinsing bath) was measured. The highest cleanliness of PN sample was reached when the surface tension of the rinsing water was that of distilled water (72 mN/m)^25^.

### Surface modification of polyester fabrics

Two-step modification of the polyester fabric surface was carried out before the immobilization of the enzyme. In the first step, either of the two different plasma treatments was used to activate the hydrophobic surface of polyester and in a second step, one of the hyperbranched poly-(ethylene glycol)-OH and poly-(amidoamine) dendrimers were grafted on plasma (both AP and CRP) treated fabrics as explained below;

***Step 1: Plasma treatments***

- ***Atmospheric pressure*** (***AP***)***plasma***
  AP treatment was carried out using a ‘Coating Star’ plasma treatment set-up provided by the Ahlbrandt System (see Fig. S2a of Supplementary Information). Our previous study described the process of atmospheric pressure plasma treatment^24–26,33,44^. Typically the fabric was displaced at a speed of 2 m/min between two electrodes (the inter-electrode distance of 1.5 mm) in an atmospheric pressure condition having a glow discharge instigated by the potential difference termed the DBD-Dielectric Barrier Discharge^33^. Plasma treatment was carried out at a power of 60 kJ/m² having a frequency of 26 kHz and was applied on both sides of PN. The generation of hydrophilic groups (carboxyl and hydroxyl) as a result of atmospheric pressure plasma treatment has already been explained in earlier studies^33^.
- ***Cold remote plasma*** (***CRP***)

Polyester fabrics were treated by cold remote plasma using the setup available at Université de Lille (France). Cleaned PN was positioned on the sample slot of the CRP device (see Fig. S2b of Supplementary Information). Gas mixtures of (N_2_ + O_2_) were excited by means of a microwave (800 W, at 2.45 GHz) to create a plasma discharge. Nitrogen gas flow was 1230 sccm and oxygen flow was 89 sccm at 3.8 mbar. The PET fabric was placed in the treatment chamber made of a quartz tube, located 90 cm from the plasma discharge, and was treated with the plasma flow at 4 mbar and 15 minutes. The plasma reaching the PET fabric is free of charged particles (ions and electrons) or UV. Due to the presence of N_2_ + O_2_ gases outspread formation of amine, imide and carboxyl groups on the surface of PET fibers can be integrated as established in existing reports^4,45,46^.

After the plasma treatments, polyester fabrics were stored in an aluminum foil to avoid aging effects before use.

***Step 2: Grafting of hyperbranched dendrimer***

- ***Grafting of poly*-**(***ethylene glycol***)**-*OH dendrimer***
  Hyperbranched poly-(ethylene glycol) pseudo generation 5 dendrimer having a hydroxyl (-OH) end group functionality (see Fig. 1a) was grafted on plasma-treated (both, atmospheric air and cold remote plasma) polyester fabric through chemical sorption method as described in our earlier studies^24,47^. Typically, plasma-activated PN was steeped into a water-ethanol 1:3 (v/v) solution having 0.30 wt.%  of dendrimer at 75 °C and stirred mildly for 5 h. The resulting materials denoted as PN@AP-PEG and PN@CRP-PEG were filtered, washed and then dried overnight at room temperature before immobilization of enzymes.
- ***Grafting of Poly*-**(***amidoamine***) ***dendrimer***

Poly-(amidoamine) or PAMAM dendrimer consists of alkyl-diamine core and tertiary amine branches (see Fig. 1b), and is the most common class of dendrimers suitable for many materials science and biotechnology applications. In this work, plasma-treated polyester fabrics were prone to chemical grafting of PAMAM. For that, PAMAM (0.3 wt.%) was chemically grafted on the plasma-treated fabrics using a mixture of ethanol/water (9:1 v/v) as a solvent at 70 °C for 24 h under N_2_ medium. The resulting materials denoted as PN@AP-PAM and PN@CRP-PAM were filtered, washed and then dried at 60 °C overnight.

### Enzyme immobilization

**Figure 2:**
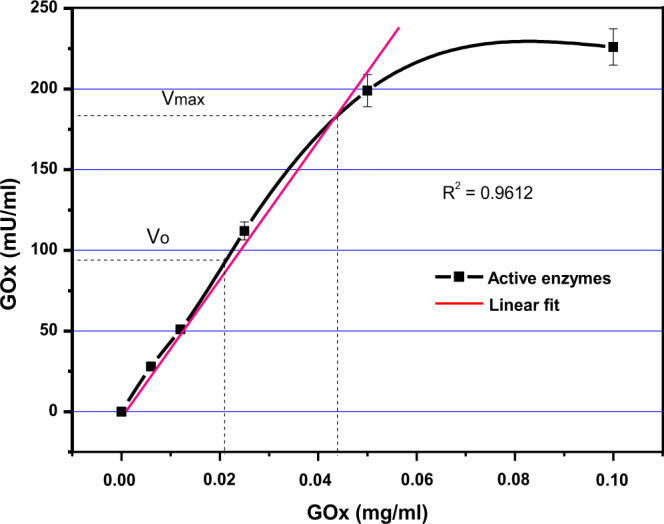
Calibration curve based on a preliminary study to identify the ideal concentration of enzymes and quantify the activity after 20 min in the presence of β-D-Glucose (500 mM). Absorbance was recorded at λ_510 nm_.

In order to ensure the uniform distribution of enzymes on surface-modified polyester fabrics, GOx was immobilized by physical adsorption method^4^. In order to choose the best condition, a calibration curve was plotted (see Fig. 2), where the maximum velocity (V_max_) of the reaction in with specific concentration of enzyme and substrates was identified. The calibration curve further provides consideration of enzyme concentration (180mU/mL) for experimental design.

Plasma treated and dendrimer grafted fabric samples (size 5 cm × 1 cm) of each type were immersed in separate enzyme solutions (pH = 7) at 4 °C for 4 h. After the immobilization process, the polyester fabrics (with immobilized enzymes) were mildly rinsed with distrilled water in a beaker for several cycles to remove the loosely attached enzymes. The rinsing water containing unfixed enzymes, and the enzyme immobilized polyester samples (after drying for 24 h at 4 °C) were preserved at 4 °C before studying the number of active enzyme units in each specimen.

### Methods

#### Methods to analyze surface modification of polyester fabrics

Contact angle and capillary uptake measurement are parameters determining the wettability of nonwovens and hence reflect the fiber surface energy and diffusion resistance, before and after modifications. The diffusional flow of reactional mixture towards and away from enzyme and an important parameter regarding biocatalytic reactions.

Wettability of the sample fabrics were analyzed through two methods; (a) sessile droplet method as described in Takke V. *et al*.^33^ was used to measure the water contact angle (${{\rm{\theta }}}_{{{\rm{H}}}_{2}{\rm{O}}}$) of more than 90° and (b) a wicking test based on capillary uptake of the specimen was concurrently chosen for ${{\rm{\theta }}}_{{{\rm{H}}}_{2}{\rm{O}}}$ < 90°. The measurements were taken by a tensiometer provided by GBX instrument (France). The analysis was carried out for a rectangular-shaped nonwovens sample of size 3 cm × 5 cm mounted on the weighing position of the tensiometer. The sample nonwovens were progressively brought into contact with the surface of distilled water placed in a container on the movable stage and measured the weight of the water entrapped inside the fabric.

After a progressive increase of weight of the sample with in the given time, the final total weight [W_t_ (t_end_)] of the sample was recorded. When the sample was separated from the water surface, the weight of water entrapped inside the nonwoven structure by capillary uptake [W_c_(t_end_)] was read directly on the screen of the balance. After that, the meniscus weight (W_m_) of the nonwovens was calculated using Eq. 1. In all the experiments, t_end_ was 3 min.

1 $${{\rm{W}}}_{{\rm{m}}}={{\rm{W}}}_{{\rm{t}}}({{\rm{t}}}_{{\rm{end}}})-{{\rm{W}}}_{{\rm{c}}}({{\rm{t}}}_{{\rm{end}}})$$

The water contact angle was calculated from the meniscus weight using Eq. 2.

2 $${{\rm{W}}}_{{\rm{m}}}\times {\rm{g}}={{\rm{\gamma }}}_{{\rm{L}}}\times \,\cos \,{\rm{\theta }}\times {\rm{\rho }}$$

Here, (W_m_) is calculated meniscus weight, (g) is gravitational acceleration, (γ_L_) is surface tension of water and (ρ) is a sample perimeter in contact with the liquid.

#### Surface analysis

Treated and untreated PN was analyzed with various techniques to study the chemical and morphological changes on the surfaces as follows;

Fourier-transform infrared spectroscopy (FTIR) of wavenumber between 400–4000 cm^−1^ was performed by a Nicolet NEXUS 670 FTIR spectrometer in transmission mode (expressed in arbitrary units) was used to study changes/integration of functional groups on the polyester surface.

X-ray Photoelectron Spectroscopy (XPS) analysis was performed by the Escalab VG220XL spectrometer (at University of Lille, France) based on a monochromatic Al Kα X-ray source at 1,486.6 eV after conditioning the samples for 15 days in ambient condition to analyze the effect of plasma treatments.

Scanning electron microscope (SEM) analysis was carried out using LEO ULTRA 55 (Carl Zeiss AG) microscope to see the changes in surface morphology of PN due to various functionalization.

#### Methods to analyze enzyme activity

The activity of enzymes (in both free and immobilized state) were studied by colorimetric assay using glucose oxidase assay kits provided by megazyme ltd. as explained in Kahoush M. *et al*.^4^. According to the principle (discussed in section 1.3 of supplementary information), GOx oxidizes β-D-Glucose to produces hydrogen peroxide as a result of the catalytic reaction. Then produced hydrogen peroxide undergoes a subsequent reaction with a secondary enzyme (horseradish peroxidase-POD) to oxidize a chromogenic substrate which results in a color change. The intensity (absorbance) of color was monitored by UV-Vis spectrophotometer at λ_510 nm_. Upon quantifying the amount of hydrogen peroxide produced by placing the absorbance difference (Δ_2_ − Δ_1_) for both blank and GOx samples in the standard curve, quantitative information of enzyme activity was obtained. Typically, 0.5 mL β-D-Glucose (500 mM) was oxidized by 0.5 mL of enzyme solution or 1 cm^2^ (out of 5 cm^2^ sample used for enzyme immobilization, see section 2.4) enzyme immobilized polyester fabric followed by simultaneous addition of 2 mL POD solution with a chromogenic substrate which gives a color change. After 20 min the absorbance of color was recorded at a fixed wavelength (λ_510 nm_) and used to quantify the number of active enzymes. The relative activity is defined as the ratio of the observed surface enzyme activity over the activity obtained from an equivalent amount of the free enzyme. All the measurements of enzyme activities were performed in 30 °C, at pH 7.

#### Quantitative analysis of enzyme immobilization

*Quantifying the amount of enzyme adsorbed:* The activity of immobilized enzymes was calculated through enzyme activity assay as explained in section 2.5.2. A detailed quantitative study about the sorption, immobilization, washed-off and denature of immobilized enzymes was carried out. The quantitative values of adsorbed enzymes on the surface of modified fabric can be determined using Eq. 3.

3 $${{\rm{E}}}_{ \mbox{-} {\rm{ads}}}=({{\rm{E}}}_{ \mbox{-} {\rm{total}}}-{{\rm{E}}}_{ \mbox{-} {\rm{remain}}})$$

Here, E_-ads_ represents the amount of enzyme deposited on the fabric, E_-total_ represents the total amount of enzyme used in the initial solution (known conc.) and E_-remain_ is the residual amount of total active enzymes remaining in the solution after immobilization.

*Quantifying the amount of enzyme washed-off during rinsing:* While part of the enzyme deposited on the fabric, was strongly adsorbed, the unfixed enzymes were rinsed out. After complete removal of the unfixed enzyme by successive rinsing, the activity of the enzyme in each rinsing water was measured. E_-wash-off_ is the sum of the total amount of active enzymes washed off during successive rinsing determined by Eq. 4.

4 $${{\rm{E}}}_{ \mbox{-} {\rm{wash}} \mbox{-} {\rm{off}}}=({{\rm{E}}}_{ \mbox{-} {\rm{rinse}} \mbox{-} 1}+{{\rm{E}}}_{ \mbox{-} {\rm{rinse}} \mbox{-} 2}+{E}_{ \mbox{-} {\rm{rinse}} \mbox{-} 3}+{{\rm{E}}}_{ \mbox{-} \mathrm{rinse} \mbox{-} 4}+{{\rm{E}}}_{ \mbox{-} {\rm{rinse}} \mbox{-} 5}+{{\rm{E}}}_{ \mbox{-} {\rm{rinse}} \mbox{-} 6\ldots \ldots \ldots }+{{\rm{E}}}_{ \mbox{-} {\rm{rinse}} \mbox{-} {\rm{n}}})$$

Here, E_-rinse-1_ is the amount of enzymes washed-off of during first rinse, E-_rinse-2, 3, 4, 5, 6…..n_ represents the amount of enzymes washed off during subsequent rinsing process.

*Reusability of enzymes and quantifying the amount of enzyme leached:* The reusability of active immobilized enzymes was studied by means of continuous assay analysis using the same experimental conditions for six consecutive cycles. After each assay, the samples with immobilized enzymes were removed from the reaction (assay) and put in a buffer solution for a few minutes followed by use for the next cycle. The activity of the immobilized enzyme was quantified using UV spectrophotometry as described in section 2.5.2. The reduction of enzyme activity after each cycle provides the characteristics of enzyme leaching (and/or denatured) into the solution. By subtracting the number of active enzymes in final polyester fabric from the initial polyester fabrics, quantitative information of leaching was estimated.

#### Antibacterial activity

The antibacterial property of GOx immobilized polyester fabric was evaluated by means of disc diffusion and zone inhibitory analysis towards gram-positive *Staphylococcus epidermidis* (ATCC 12228) and gram-negative *Escherichia coli* (ATCC 25922) bacteria strains. The antibacterial behavior of the biocatalyst was measured on individual bacteria type. Mueller Hinton agar plates were used for diffusion and zone inhibition analysis, the test is a semi-quantitative method where the antibacterial activity was assessed by examining the absence or presence of microbial growth in the contact zone between agar and specimen and on the eventual appearance of an inhibition zone according to ISO 20645. The catalyst of dimension 1 cm × 1 cm was placed on the surface of the nutrient agar medium after soaking in a glucose solution (500 mM) which was swabbed with the bacterial (106 CFU/cm^3^) culture. The plates were incubated at 37 °C for 24 hours to measure the zone of inhibition in millimeters formed around the catalyst. The results are expressed as the width of the inhibition zone (mm).

## Results

Table 1 summarizes the terminology and corresponding description of treatment for individual samples studied in this work. The samples were denoted in further texts according to their chosen acronyms.

**Table 1:** Terminology of samples.

Sample acronym	Treatments		
	Plasma	Dendrimer	Enzyme
PN@AP/GOx	Atmospheric pressure (AP)	—	Glucose oxidase
PN@CRP/GOx	Cold remote plasma (CRP)	—	Glucose oxidase
PN@AP-PEG/GOx	Atmospheric pressure (AP)	Hyperbranched poly-(ethylene glycol)-OH	Glucose oxidase
PN@AP-PAM/GOx	Atmospheric pressure (AP)	Poly-(amidoamine)	Glucose oxidase
PN@CRP-PEG/GOx	Cold remote plasma (CRP)	Hyperbranched poly-(ethylene glycol)-OH	Glucose oxidase
PN@CRP-PAM/GOx	Cold remote plasma (CRP)	Poly-(amidoamine)	Glucose oxidase

### Part 1- Analysis of surface modification of polyester fabrics

#### Analysis of polyester surface activation by plasma treatments. Contact angle and capillary uptake analysis:

A sessile water droplet deposited at the nonwoven surface was observed and measured using a digital camera to study the water contact angle (${{\rm{\theta }}}_{{{\rm{H}}}_{2}{\rm{O}}}$) at the polyester surface before and after plasma treatment. It showed that (see Fig. S3 in Supplementary Information), the water contact angle (${{\rm{\theta }}}_{{{\rm{H}}}_{2}{\rm{O}}}$) was as high as 141° at the surface of untreated polyester fabric, which is higher than that of standard polyester fiber surface (${{\rm{\theta }}}_{{{\rm{H}}}_{2}{\rm{O}}}$ = 80°). The high surface roughness due to irregularities of the fabric surface would account for such a high water contact angle. However, it was further found that, upon plasma treatment, the droplet was immediately absorbed (see Fig. S3-b in supplementary information), and ${{\rm{\theta }}}_{{{\rm{H}}}_{2}{\rm{O}}}$ calculated from the meniscus weight [Eq. (1)] was 0° for both PN@AP and PN@CRP.

Fig. S4 (in Supplementary Information) shows the capillary uptake performance of untreated and plasma-treated polyester fabrics. Due to hydrophobic surface groups of polyester surface, untreated polyester showed no capillary rising (a slight uptake as shown in Fig. S4 is due to the weight of the meniscus). On the other side, atmospheric pressure and cold remote plasma treatments introduce hydrophilic carboxyl groups (–COOH), amine and amide groups on the polyester fabric surface. Due to those functional groups, plasma treated polyester fabrics showed significant capillary uptake to be considered as hydrophilic. However, between two different plasma treatments, higher capillary uptakes (1460 mg) was measured on AP treated fabrics compare to CR plasma (919 mg) treated fabrics.

#### XPS analysis

The addition or changes in chemical functional groups occurring on the polyester fabric due to plasma treatment has been detected by X-ray Photoelectron Spectroscopy (XPS) analysis (see Fig. S5 in supplementary information). Table 2 summarizes the elemental compositions of untreated and plasma-treated polyester surfaces. Atomic ratio O/C = 0.3 has been recorded for untreated polyester fabric, which increased to 0.5 upon atmospheric pressure plasma treatment can be due to the generation of chain scissions at ester bonds with the integration of O and formation of carboxyl and hydroxyl terminal groups as consistent with our past studies^24,25^. The spectra detected the existence of N for PN@CRP, which is due to the presence of gas used during the treatment. As can be seen, O/C ratio, initially for untreated PN (0.3) increased to 0.38 along 0.6% of N in PN@CRP providing evidence for the formation of the polar terminal group as well as amine and amide groups, consistent to the literature^4^.

**Table 2:** Physico-chemical properties of polyester fabric before and after plasma treatment.

Samples	Water contact angle	Capillary uptake (mg)	Chemical functional groups (XPS analysis)
PN (Untreated polyester fabric)	141°	—	O/C = 0.3
PN@AP (Polyester surface modified by atmospheric pressure plasma)	0°	1460	Carboxyl groups (COOH) Hydroxyl groups (OH) N = 0% O/C = 0.5
PN@CRP (Polyester surface modified by cold remote plasma in presence of N_2_ + O_2_)	0°	919	Carboxyl (COOH) and amino (–NH_2_) groups O/C = 0.38 N: 0.6%

#### FTIR chemical analysis

Fourier transform infrared spectroscopy (FTIR) analysis was carried out to obtain the transmittance spectrum of untreated and plasma-treated polyester fabric are illustrated in Fig. S6 in the Supplementary Information. It can be seen that spectra of untreated polyester fiber were not showing any significant feature whereas newly integrated oxygenated polar functional groups and amino functional groups were in existence on the spectrum of plasma (AP and CRP respectively) activated PN. Strong peaks of ether (950–1200 cm^−1^), carboxyl and carbonyl groups (1650–1710 cm^−1^) were observed on spectra for all samples. Peak present in all samples at 1733 cm^−1^ is the stretching vibration of C=O of PET^45^. On the other hand in PN@AP, several weak peaks appearing in the region at 3700–3684 cm^−1^ was attributed to O–H stretching can be due to the integration of new carboxyl groups. Along with similar stretching, weak stretching at region 3346 cm^−1^ observed in PN@CRP was assigned to the stretching vibration of both carboxyl and secondary amine groups^4^. Characteristics double peaks at 2830 cm^−1^ and 2960 cm^−1^ region were assigned for the stretching vibration of =C–H of aldehyde doublet.

#### Analysis of polyester fabric surface after dendrimer grafting

**Figure 3:**
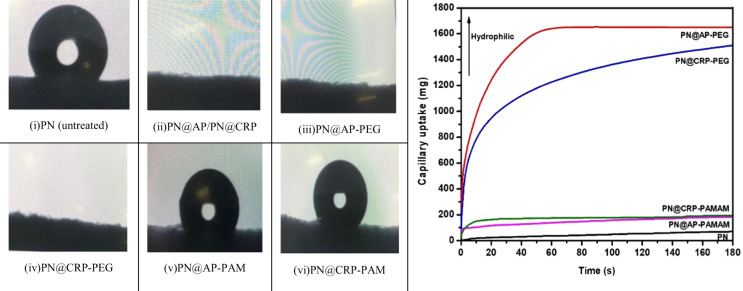
Contact angle (on left) and capillary uptake measurement analysis (on right) of untreated and dendrimer grafted polyester fabrics.

Contact angle and capillary uptake analysis: Change in wettability of plasma-treated polyester fabrics due to further integration of polar and nonpolar functional groups through dendrimer grafting was studied by means of contact angle and capillary uptake analysis. A major change in the wettability of the plasma-treated fabrics were detected as shown in Fig. 3. A significant decrease in capillary uptake (corresponds to increase in contact angle) on PAMAM dendrimer grafted PN (compared to PEG-OH) can be due to the high integration N as amino groups without having any polar groups which are in agreement with the report by Mutel *et al*.^48^ and Arfaoui *et al*.^25^. This phenomenon proved that indeed the PAMAM dendrimer was grafted successfully on the fiber surface.

On the other side, an increase in wettability of PN@CRP and PN@AP as referring to Fig. 3 indicates the presence of more hydrophilic polar groups, which is certainly evidence of the grafting of hyperbranched PEG–OH dendrimers. Both of these groups are favorable for improvement in adhesion between the external matrix and fabric surface.

#### Surface analysis

**Figure 4:**
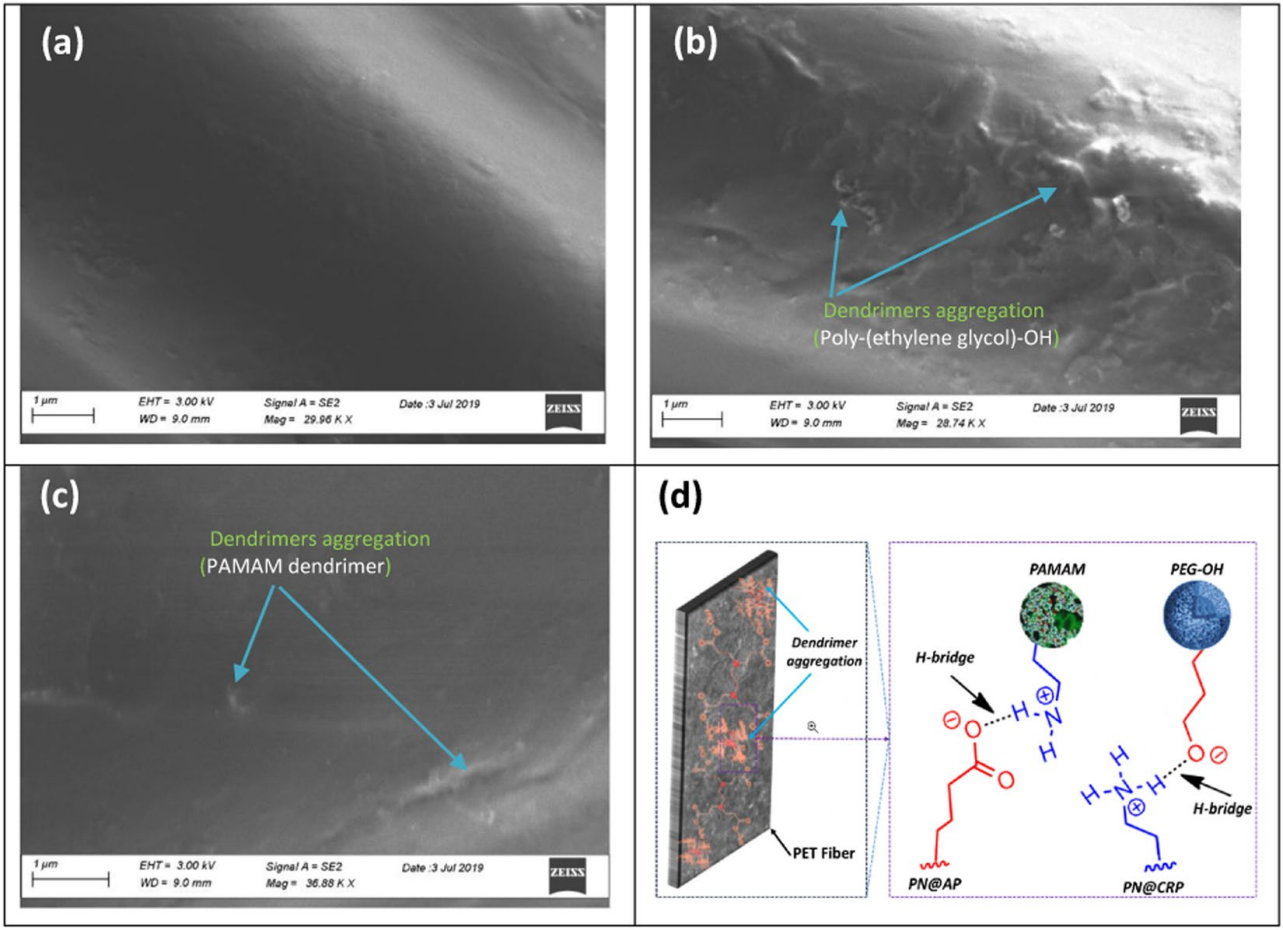
SEM: changes in surface morphology of the polyester fabric upon dendrimer grafting; (**a**) PN, (**b**) PN@AP-PEG, (**c**) PN@AP-PAM and (**d**) schematic illustration of incorporation of dendrimer on plasma-activated PET surface.

Characteristics surface morphological changes of polyester fabric due to the grafting of hyperbranched dendrimers were studied through a scanning electron microscope (SEM) as shown in Fig. 4. Untreated PET fiber surface showed a smooth surface (see Fig. 4a). Dendrimer incorporation induced a marked flattening of the fiber surface, presumably due to dendrimer aggregation via H–bridges. This is supposed to produce a more disordered arrangement and morphology changes of the material, as shown in Fig. 4b–d. This change was more pronounced for the PEG–OH as compared to the PAMAM dendrimer, and can be due to their chemical moiety and state of matter. The relatively plain morphological surface indicates a more uniform dispersion of the dendrimers within fibers interface and on the fiber surface. However, the quasi-uniform morphological surface must be due to dendrimer aggregation. This result is of great importance since it clearly demonstrates that atmospheric plasma treated polyester nonwoven promotes dendrimer dispersion, indicating a significant depletion of accessible –OH groups. This also clearly demonstrates that the plasma-treated nonwoven surface plays a key role in promoting competitive H–bridges with the polyol molecules that prevent aggregation and favor dendrimer dispersion on the surface.

### Part 2- Analysis of immobilization of GOx enzyme on modified polyester fabrics

#### Yields of GOx enzyme loading on polyester fabrics

**Figure 5:**
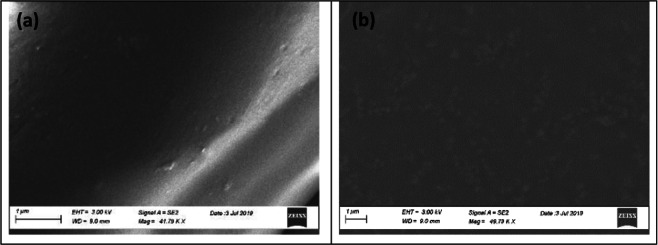
SEM images of the surface of (**a**) untreated polyester fiber and (**b**) GOx immobilized polyester fiber (PN@CRP-PAM).

The direct role of enzymatic catalysis using immobilized enzymes on porous textiles is subjected to the dispersion of enzymes on that support material. Better dispersion offers competent catalytic behavior, though the accessibility of the enzyme active site by the substrate is an important parameter, too. The effect of two different plasma treatment and dendrimers used to functionalize polyester fabric towards loading/dispersion of GOx enzymes were tested. Solution concentration (180 mU/mL) of enzymes was the same for all the samples. Qualitative analysis by scanning electron microscope reveals randomly oriented and solely dispersed bright dots of immobilized enzymes on the surface-modified polyester fibers (see Fig. 5b) whereas untreated polyester shows a fairly smooth surface (see Fig. 5a).

**Figure 6:**
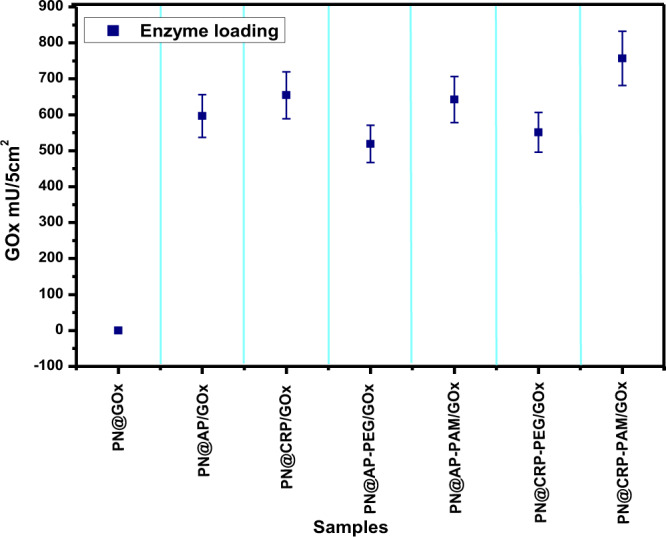
Relative loading of GOx enzymes as a function of surface treatment of the polyester fabrics.

The quantitative analysis of enzyme loading on each nonwoven was studied as per the method explained in section 2.5.3, and displayed in Fig. 6. Here the adsorption of enzymes on 1 cm^2^ functionalized polyester fabric was calculated and relative enzyme activity of 5 cm^2^ samples was estimated and compared as per Eq. (3) (see section 2.5.3). It was found that, the polyester fabrics having amine/imide/amide end functional groups provided better enzyme activity compared to fabrics with hydroxyl end-functional groups, this can be due to the attraction between two amino acids when the carboxylic acid group of one amino acid reacts with the amine group of the other amino acid to form a peptide link as explained by Eyre, David *et al*.^49^. The highest enzyme adsorption/loading (757 mU/5 cm^2^) was found for PN@CRP-PAM designates the effectiveness of CRP and PAMAM dendrimer.

On the other hand, least loading was observed for PN@AP-PEG followed by PN@CRP-PEG, which indicates the low affinity of enzymes towards hydroxyl functional groups as confirmed in our previous reports^25^. Interestingly PN@AP-PAM and PN@CRP, which showed similar loading efficiency provide the evidence for analogous surface features.

**Figure 7:**
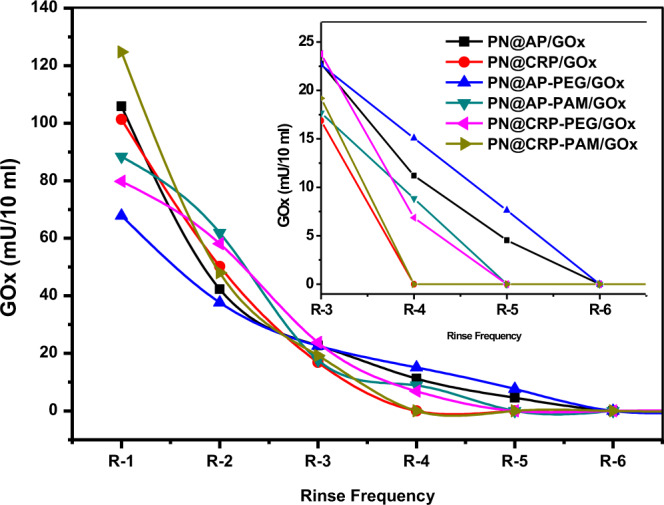
Removal of unfixed enzymes as a function of rinse frequency.

The influence of integrated functional groups on PN:GOx complex has been studied as a function of rinse frequency after immobilization. Successive rinsings of the freshly immobilized polyester fabrics removes loosely attached enzymes from the fiber surface. Results in Fig. 7 show that up to six (06) rinsings are required to remove all loosely attached active enzymes. However, enzymes immobilized on PN@CRP/GOx and PN@CRP-PAM/GOx secure the further loss of enzymes after four rinseings. Quantitative analysis of the total amount of active enzymes washed-off from the fabric surface during each rinsing is summarized in Table 3, which further provides a decisive measure of enzyme loading.

**Table 3:** Quantification of the amount of enzyme washed-off during rinsing.

Sample	Total enzyme washed away during rinse (mU/5 cm^2^)^a^	% of enzyme washed away during rinse^b^	Actual loading of enzymes (mU/5 cm^2^)^c^
PN@AP/GOx	211	35	385
PN@CRP/GOx	168	25	485
PN@AP-PEG/GOx	150	29	368
PN@AP-PAM/GOx	176	27	465
PN@CRP-PEG/GOx	157	28	394
PN@CRP-PAM/GOx	191	23	565

Note: ^a^Total washed-off active enzyme was the addition of all active enzymes washed during each rinse.

^b^Percentage of active enzyme washed away was calculated against the immobilization of active enzyme.

^c^The actual loading of enzymes were calculated by subtracting the total amount of enzyme washed away during rinse from initial loading.

However, the polyester fabrics functionalized with amine terminal functional groups lead to high loss of enzyme during rinses, also provide robust enzyme loading. This can be seen in the arithmetic analysis of % of enzyme washed away during the rinses. The amine-functionalized polyester loses 23–27% of active enzymes where fabrics having hydroxyl functional groups lose 28–35% of adsorbed enzymes after 6 rinsing proves the claim that, polyester fabric functionalized with CR plasma and/or PAMAM dendrimer provides better enzyme immobilization performance. The quantitative analysis of the actual loading of enzymes further supported the claim through a similar trend. The phenomena are the opposite of what it was on loading, which can be explained by the high tendency of enzyme affinity towards amine groups (see Table 3). The higher tendency leads to higher adsorption of the enzyme on the fiber surface through different electrostatic bonds providing a high amount of loosely attached enzymes. On the other side, the low affinity of hydroxyl groups results in a fewer amount of loosely attached enzymes on their surface. However, an exception on characteristics has been observed in PN@AP-GOx sample, where the highest amount of enzymes (among all samples) found to be washed-off during rinsing. This can be attributed to the presence of carboxyl groups along with hydroxyl groups integrated through plasma treatment^25^.

#### Yields of the active immobilized enzyme on polyester fabrics

**Figure 8:**
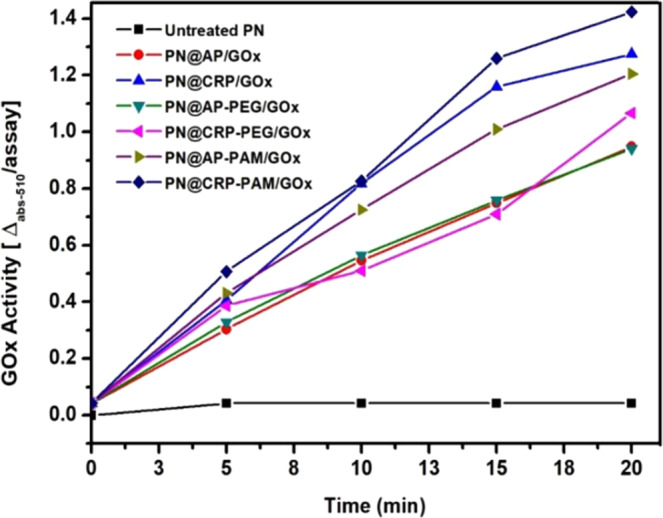
Yields of the concentration of active enzyme as a function of time for differently functionalize polyester fabrics.

The real-time activity of immobilized enzymes was measured for all the bio-functionalized polyester using the glucose oxidase activity kit according to the method described in section 2.5.2. The enzyme activity was measured for 20 min according to the protocol provided with the kits as explained by Kahoush M. *et al*.^4^. Results displayed in Fig. 8 shows a linear increase in GOx activity as a function of time. It must be noted that, when the amount of enzyme is fixed, the reaction rate can be increased with the increase of substrate in a nonlinear approach, so, if activities are measured over longer contact times, the final activity may be found to be reduced per unit time^50^. The activity can be tentatively explained by the interplay of several factors: concentration of glucose, the concentration of glucose: GOx complex, the concentration of product (D-glucono-δ-lactone and hydrogen peroxide H_2_O_2_).

A constant enzymatic reaction condition was selected and used for all the samples for precise contrast and comparison. As can be seen (see Fig. 8), with the untreated polyester fabric, no significant enzymatic activity was present. Results summarized in Table 4 shows that all the surface-modified polyester fabric carried a sufficient percentage (62–81%) of active immobilized enzymes. However, different functionalization offers insight into the effectiveness of modification on immobilization efficiency.

**Table 4:** Quantification of the amount of active enzyme immobilized on surface-modified polyester fabrics.

Sample	% of active immobilized enzymes
PN@GOx	—
PN@AP/GOx	62
PN@CRP/GOx	74
PN@AP-PEG/GOx	68
PN@AP-PAM/GOx	78
PN@CRP-PEG/GOx	70
PN@CRP-PAM/GOx	81

Note: The % of active immobilized enzymes were calculated by colorimetric analysis of the 1 cm^2^ sample.

As consistent to the previous analysis (in section 3.3) on enzyme loading, amine functional groups integrated polyester fabrics provided highest 81% (PN@CRP-PAM/GOx) active immobilized enzymes followed by and PN@CRP/GOx (74%) whereas hydroxyl functional groups integrated samples showed 62% and 68% for PN@AP/GOx and PN@AP-PEG/GOx respectively. Samples having both amine and hydroxyl groups showed the loading of 78% and 70% active immobilized enzyme for PN@AP-PAM/GOx and PN@CRP-PEG/GOx respectively. An interesting phenomenon has been noticed for both amine/hydroxyl intergraded samples is that, even though the samples were treated for both amine and hydroxyl surface functional groups, their ability to immobilize active enzymes has been influenced by the order of the surface treatment. Indeed the percentage of active enzymes on PN@AP/GOx is equitable, yet the lowermost amount of immobilized active enzymes maintained their bio-catalytic activity after immobilization.

In order to evaluate the effect of immobilization on the kinetic parameters of the enzymatic reaction, the parameters were calculated for the in free and immobilized GOx. The initial velocities of experiments conducted at different glucose concentrations (2 mM to 50 mM) were evaluated using the Michaelis-Menten model of enzyme kinetics^51^ and the Lineweaver-Burk method was used to estimate the apparent Michaelis constant (K_m_) and maximum velocity of the reaction (V_max_) as summarized in Table 5.

**Table 5:** Kinetics parameters of free and immobilized GOx.

Sample	V_max_ (mM min^*−*1^)	K_m_ (mM)
Free GOx	2.86	5.80
PN@AP/GOx	1.52	7.11
PN@CRP/GOx	2.10	6.38
PN@AP-PEG/GOx	1.38	8.11
PN@AP-PAM/GOx	1.98	6.75
PN@CRP-PEG/GOx	1.68	6.47
PN@CRP-PAM/GOx	2.21	6.16

Michaelis–Menten equation [Eq. (5)] as follows,

5 $${\rm{Reaction}}\,{\rm{rate}},\,{{\rm{V}}}_{0}={{\rm{V}}}_{{\rm{\max }}}[{\rm{S}}]/{{\rm{K}}}_{{\rm{m}}}+[{\rm{S}}]$$

K_m_ and V_max_ were determined by incubating the GOx (free and immobilized) with varying concentrations of the substrate; the results can be plotted as a graph of rate of reaction against concentration of substrate, and will normally yield a hyperbolic curve, as shown in the Fig. S7 of supplementary information. The standard curve relating to glucose oxidase activity (mU/assay i.e. /0.5 mL) to absorbance at λ_510 nm_ has been shown in Fig. S8. As indicated in the table, the immobilized GOx showed a decrease in V_max_, and an increase in the value of K_m_ for all immobilized enzyme as compared to the free GOx (V_max_ = 2.86 mM min^−1^, K_m_ = 5.80 mM). The increase in the value of K_m_ could be attributed to the lower accessibility of the active sites of immobilized GOx to the glucose molecules. The decrease in V_max_ value as a result of immobilization is considered to be associated with the lower affinity of the substrate to the enzyme active site. However, on the overall, minimum reduction in V_max_ as well as minimum increase in K_m_, were observed for PN@CRP-PAM/GOx (Table 5), which again confirm the highest enzymatic catalytic activity of enzyme immobilized on polyester nonwovens functionalized by both CRP plasma and PAMAM dendrimer.

#### Leaching of the active immobilized enzymes as a function of reusability cycles

**Figure 9:**
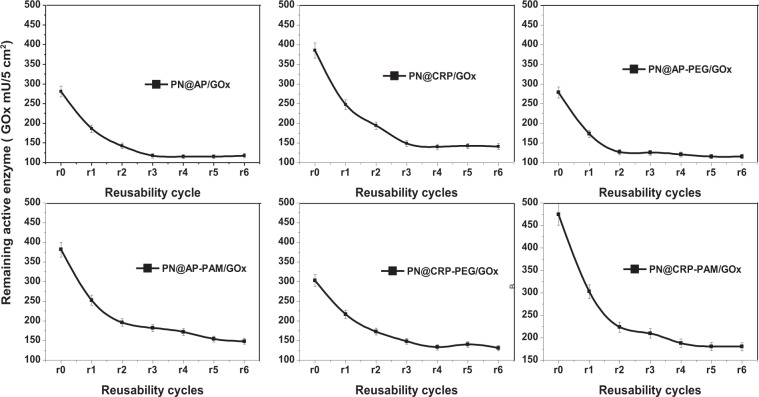
Reusability analysis of individual polyester fabric as a function of enzyme activity.

The stability of immobilized enzymes and the reproducibility of these samples were studied through the activity of enzymes after each reusability cycle. The enzyme reusability study as shown in Fig. 9 was carried out according to the method described in section 2.5.2, where all the parameter remains constant for each cycle analysis.

It can be seen from the results that, the enzymatic activity of all samples showed a sharp decreased in activity on the first three reusability cycles, which later showed a moderate decrease to five cycle applications followed no significant decrease in enzyme activity after 6 cycle application. The summary of enzyme leaching on Table 5 shows lowest 37% leaching/inhibition of immobilized enzymes on PN@CRP/GOx sample, whereas PN@AP/GOx showed leaching/inhibition of almost 50% enzymes after 6 cycle application.

#### Discussions for catalytic activity improvement of immobilized enzymes

This study shows that both plasma treatment and dendrimer grafting effectively activate the hydrophobic surface of polyester fabric. The functionalized polyester fabrics were used to immobilize the glucose oxidase enzyme resulted in a polyester fabric with bio-catalytic activity. However, the effect of two different plasma treatments and two different dendrimers shows a significant difference in surface properties and enzyme immobilization. Wettability analysis summarized the effects of different treatments on the surface roughness and capillary uptake of the polyester nonwovens. Due to the surface activation, there was a significant decrease in water contact angle, corresponding to an increase in hydrophilicity of the fabrics. Primarily, the radicals formed during the plasma treatment due to the scission of ester bonds generate a polar surface with end-functional groups such as C–O, C=O, C=N, N–H and N–C–O which was confirmed by the FTIR analysis. Indeed addition of O_2_ in CRP plasma treatment produces polar terminal groups that give PN@CRP a hydrophilic surface whereas the presence of N_2_ explained by the formation of amine, imide and amide groups on the polyester surface. These polar groups integrated on plasma-treated polyester allow the formation of dipolar interactions, van der Waals forces, or hydrogen bonds between the fabric and dendrimers. Upon grafting of dendrimers, the polyester fabrics could be divided into two groups, one having hydroxyl functional end groups whereas the other one contains amine functional end groups. Both functional groups provide favorable conditions for enzyme immobilization^52,53^.

**Figure 10:**
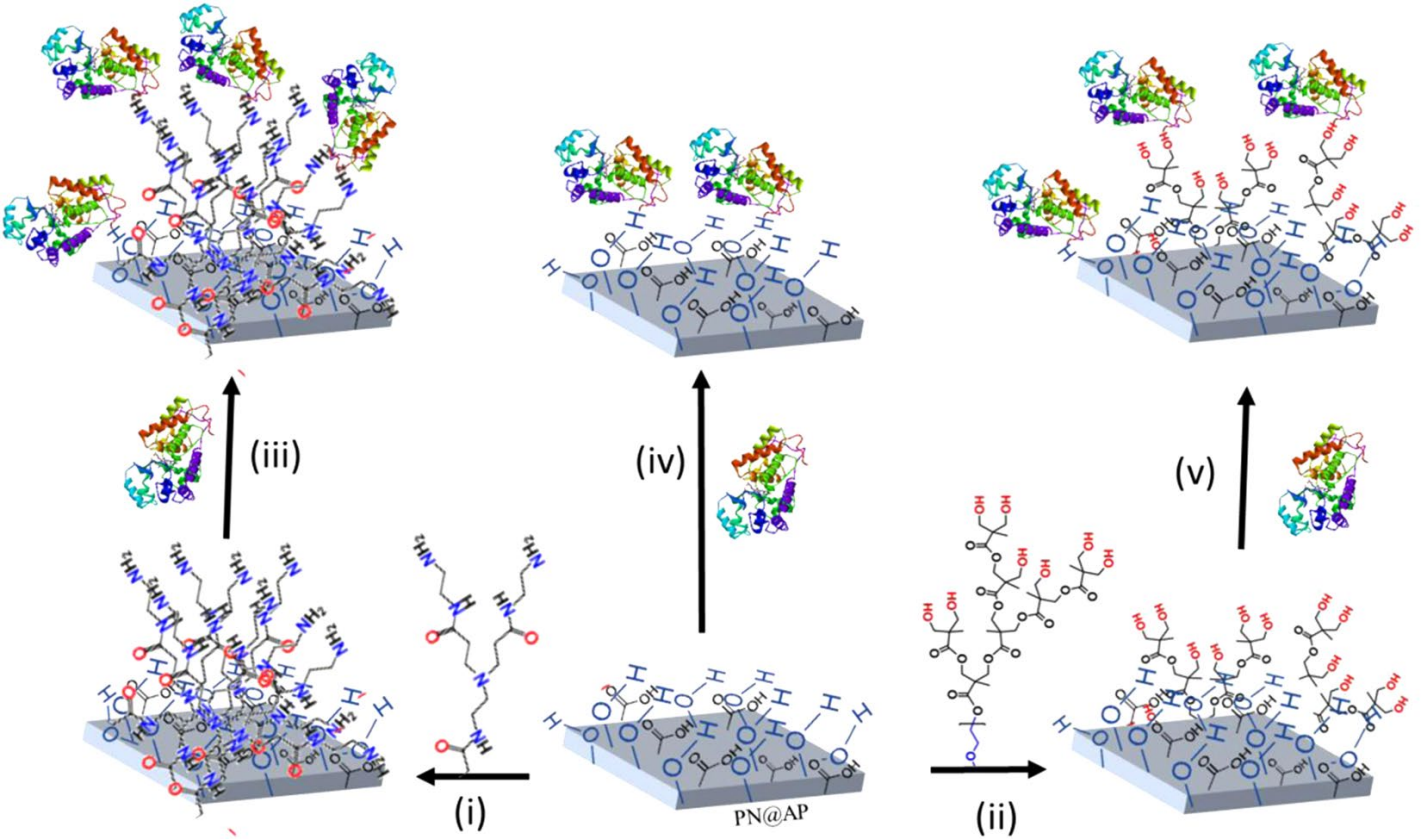
Schematic illustration of dendrimer grafting and immobilization of glucose oxidase enzymes on plasma-treated polyester fabrics; (i) grafting of PAMAM dendrimer, (ii) grafting of hyperbranched PEG–OH dendrimer, (iii) enzyme immobilization on PAMAM dendrimer grafted polyester fabric, (iv) enzyme immobilization on atmospheric pressure plasma treated polyester fabrics and (v) enzyme immobilization on PEG–OH dendrimer grafted polyester fabrics.

The loading of GOx enzymes on the polyester surface was influenced by the functional groups integrated by plasma treatment and dendrimer grafting. Amino acids are organic compounds containing amine (–NH_2_) and carboxyl (–COOH) functional groups are the sole element of all known enzymes^54–56^. So, this is apparent that amino groups would provide the most favorable condition towards a robust enzyme immobilization. Since polyester fabric was functionalized by plasma/dendrimer (amine/hydroxyl), enzymes covalently coupled with the polyester fabric by reacting with corresponding functional groups as illustrated in Fig. 10. Results showed that, between hydroxyl and amino groups, amino groups provide better enzyme immobilization efficiency. Active sites of amine functional groups brought a high amount of enzymes to the fiber surface. In a pattern where the amount of estimated amine function groups is higher, higher enzyme affinity was shown.

In addition to the creation of hydrogen bonding with carbonyl groups, possible covalent bonding with the amino acids of the enzymes^57,58^ can lead to less leaching of the enzyme into the reaction medium as summarized in Table 6.

**Table 6:** Summary of enzyme loading, immobilization, and leaching.

Sample	% of enzymes adsorbed	% of active immobilized enzyme	% of leached active immobilized enzyme
PN@AP/GOx	21	62	48
PN@CRP/GOx	27	74	37
PN@AP-PEG/GOx	20	68	42
PN@AP-PAM/GOx	26	78	39
PN@CRP-PEG/GOx	22	70	41
PN@CRP-PAM/GOx	31	81	38

The less leaching of enzymes consequently extends the reusability of immobilized enzymes. Additionally, the PET nonwoven is inherently a 3D porous material composed of cylindrical fibers and with high porosity. While high porosity can allow easy inflow and outflow of reactional mixtures (substrate, or products), the total surface area of fiber surface per unit area of nonwoven material is high enough to allow fixation of sufficient amount of enzymes. Hydrophilisation of PET surface via plasma and/or dendrimer grafting would not only improve this flow of liquid, but would also create specific functional groups, which allow appropriate immobilization of enzyme with accessibility to the active site, and hence improved bioactivity.

#### Effect of temperature on the relative activity of free and immobilized enzymes

**Figure 11:**
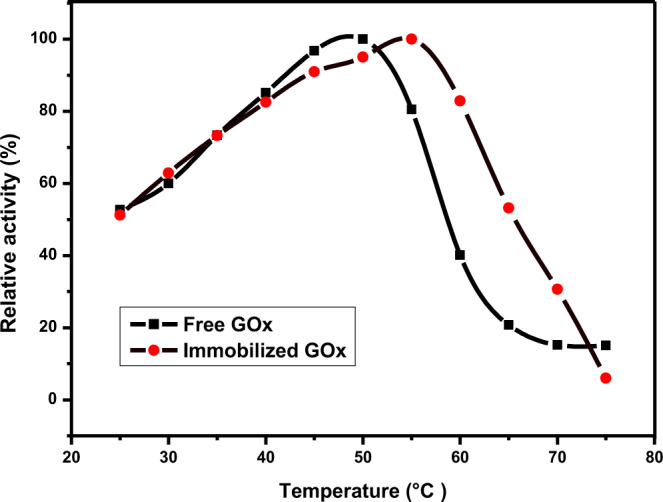
Effect of temperature on bio-catalytic activity immobilized (PN@CRP-PAM/GOx) and free enzyme (200 mU/mL). The relative activity (maximum active immobilized enzymes found on primary samples corresponding to 100% of relative activity) obtained for each temperature used with free and immobilized GOx.

The impact of temperature on bio-catalytic activity immobilized (PN@CRP–PAM/GOx) and free enzyme (200 mU/mL) was comparatively studied. Enzyme activity was measured at different temperatures from 25 °C to 75 °C. In order to better compare the behavior of free and immobilized GOx, data were normalized with respect to the highest value of activity displayed by each form of the enzyme. The relative activity (maximum active immobilized enzymes found on primary samples corresponding to 100% of relative activity) obtained for each temperature used with free and immobilized GOx is plotted in Fig. 11. The graph shows a slight shifting of the enzyme activity optimal temperature between the free and immobilized conditions. Free GOx displayed a maximum activity at 50 °C which decreased by 50% at ~60 °C and continued to decline until the activity is absent at 75 °C indicating enzyme denature due to temperature-related destruction of the enzyme structure as consistent to the literature^59,60^. In contrast, immobilized GOx reaches the highest activity at 55 °C which decreased by 50% at ~65 °C indicating an increase in improvement of thermal stability of the immobilized enzyme. The sharp decrease in the activity of immobilized GOx can be correlated to the denaturation of enzyme at high temperature as well as glass transition temperature (Tg) of PN (support material of immobilized GOx) which is about 70 °C^25^. The progressive increase in activity of the immobilized enzyme (at neutral pH) provides evidence for the lasting potential of the enzymatic reaction, which will further ensure better reaction control over free enzyme for industrial practices.

## Antibacterial Activity of Enzyme Immobilized Polyester Fabrics

As prospective, the antibacterial properties of enzyme immobilized polyester fabrics were investigated under ambient conditions. The inhibition zone of PN@AP/GOx, PN@CRP/GOx, PN@AP-PEG/GOx, PN@AP-PAM/GOx, PN@CRP-PEG/GOx, and PN@CRP-PAM/GOx was measured for two bacteria (*E*. *coli* and *S*. *epidermidis*), and the results showed that the functional nonwovens effectively inhibited the bacterial growth (see Fig. S9 of Supplementary Information). This was the first overview to confirm the antibacterial activity of immobilized glucose oxidase enzyme on a fibrous textile. A blank experiment has been conducted using untreated PN. There was no inhibition zone noticed for untreated PN, which means PN fibers do not have antibacterial behavior without additional antibacterial agents. However, upon immobilization of the enzyme on PN, all functional nonwovens effectively inhibited bacterial growth, and the inhibition zone varied from 19 mm to 41 mm according to the functionalization method used for enzyme immobilization. According to ISO 20645 standard, the outspreading up to 1 mm inhibition zone and no growth under specimen or no inhibition zone is accepted as effective. This result is of great importance and can be explained by the strong capacity of hydrogen peroxide (produced due to the catalytic oxidation of glucose) to alter the membrane of *E*. *coli* and *S*. *epidermidis*, inducing cell wall rupture. The zone inhibition diameter is summarized in Table 7.

**Table 7:** The zone of inhibition* (mm) analysis of catalysts according to test method ISO 20645.

Catalysts	*Staphylococcus epidermidis*	*Escherichia coli*
Untreated PN	—	—
PN@AP/GOx	19 mm	23 mm
PN@CRP/GOx	21 mm	29 mm
PN@AP-PEG/GOx	31 mm	33 mm
PN@AP-PAM/GOx	38 mm	38 mm
PN@CRP-PEG/GOx	39 mm	38 mm
PN@CRP-PAM/GOx	41 mm	35 mm

*Mean values were given in the table after performing the test three times for each sample.

## Conclusions

In summary, robust grafting of glucose oxidase enzyme on polyester surface modified through plasma and hyperbranched dendrimers has been achieved. The synergistic effect of hydroxyl, carboxyl, amine and amide groups enabled immobilized enzymes on a textile surface to remain active, maintaining stability and reusability properties as summarized below:

- Resultant surface-modified polyester showed sufficient presence of surface-active groups upon plasma treatment and dendrimer grafting, confirmed by wettability, SEM and XPS analysis.
- Enzyme activity analysis reveals that modified polyester having amine terminal functional groups provides the most favorable condition for enzyme immobilization providing, up to 31% enzyme loading, and 81% activity of immobilized enzymes.
- Robust immobilization of glucose oxidase enzymes may be explained by covalent interaction of amine groups and carboxyl groups present in GOx and modified PN as seen by colorimetric assays and successive reusability of the enzyme immobilized catalysts.
- The first overview to confirm the antibacterial activity of immobilized glucose oxidase enzyme on a fibrous textile showed high antibacterial activity (zone inhibition up to 41 mm).
- Potential improvement in catalytic reaction rate suggests a significant prospect to use PN as a robust textile capable of bio-catalysis on the heterogeneous catalytic system.

This study concludes novel approaches for improvement of enzyme immobilization efficiency by modifying the surface of polyester using sustainable tools and provides a foundation for the further rational design and technical background for an efficient catalyst for various bio-industrial applications.

## Supplementary information

Supplementary Information
