# Supplementary material for: Surface modification of polyester fabric using plasma-dendrimer for robust immobilization of glucose oxidase enzyme
Source: Sci Rep. 2019 Oct 31;9:15730. doi: 10.1038/s41598-019-52087-8 (PMC6823486; doi:10.1038/s41598-019-52087-8)
Supplement: Supplementary file 1 — Supplementary Information [file 41598_2019_52087_MOESM1_ESM.pdf]

-----Supplementary information-----

Surface modification of polyester fabric using plasma-dendrimer for robust immobilization of glucose oxidase enzyme

Mohammad Neaz Morshed [1, 2, 3, 4*], Nemeshwaree Behary [2, 3], Nabil Bouazizi [2, 3], Jinping Guan[4,] Guoqiang Chen[4] and Vincent Nierstrasz [1]

[1] Department of Textile Material Technology, The Swedish School of Textiles, Faculty of Textiles, Engineering and Business, University of Borås, SE-50190, Borås, Sweden
[2] Ecole Nationale Supérieure des Arts et Industries Textiles (ENSAIT), GEMTEX Laboratory, 2 allée Louise et Victor Champier BP 30329, 59056 Roubaix, France
[3] Université de Lille, Nord de France, F-59000 Lille, France
[4] College of Textile and Clothing Engineering, Soochow University, 215006 Suzhou, China
* **Corresponding authors:** mohammad_neaz.morshed@hb.se (M.N Morshed);

1. MATERIALS AND METHODS

1.1. Polyester fabric

Polyester fabrics were cleaned in order to remove impurities and spinning oil present on the surface as described in our previous work [1-4]. Typically, PET nonwovens were cut into 210 mm x 297 mm and engrossed into petroleum ether solution using Soxhlet (see **Figure S1**) for 5 hours at 60°C. In the next step nonwovens were ultrasonically rinsed along with absolute ethanol for 20 min followed by drying overnight. After that, nonwovens were repeatedly ultrasonically washed 3 times with deionized water and dried. The cleaned nonwovens were preserved for pre-activation. The characteristics of nonwoven are displayed in **Table S1.**

Tables S1: Characteristics of PET nonwovens

Sample/characteristics	Values
Mass per unit area (g/m2)	98.00
Thicknesses (mm)	0.94
Fiber density	0.80
Porosity (%)	99.91
Air permeability (mm/s)	854.20

1.2. Plasma treatment of polyester fabrics

Plasma treatments modify polymer surfaces using plasma gases made up of a mixture of charged particles (electrons and ions), excited species (free radicals, meta-stable molecules), and photons. Atmospheric air plasma treatment

device installed in The École nationale supérieure des arts et industries textiles (ENSAIT) France was used to treat the samples in a continuous treatment process. (see **Figure S2a**) [5].

An experimental setup available at university of science and technology of Lille, France was used for plasma treatment (see **Figure S2b**). In this process, gaseous flow ($N_2 + O_2$) provided by a continuous pumping with rotary pump (33 m^3/h). It was excited by an electrodeless discharge provided by a microwave generator (2450 MHz) with a capacity of delivering a transmitted power up to 1200 W. The discharge was produced in a quartz tube (inner diameter 30 mm) coupled to the Pyrex cylindrical treatment chamber (diameter 150 mm and volume 15 L) [6]. The distance between the discharge and the treatment zone was 900 mm. The gaseous flow was controlled by means of a mass flow regulator and the pressure was measured with a Pirani gauge.

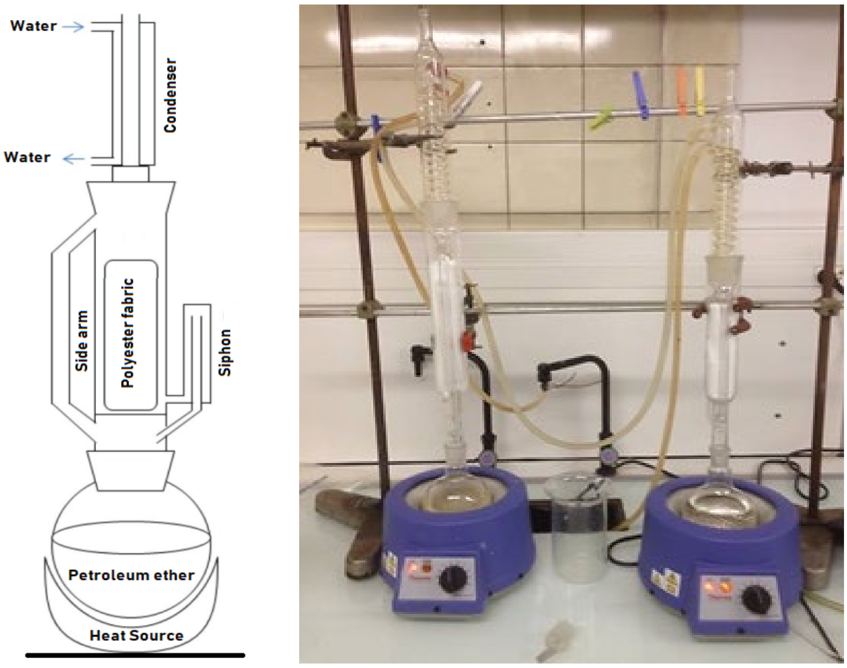

Figure S1: Schematic illustration and digital photograph of cleaning of polyester fabric using Soxhlet.

(a) **(b)**

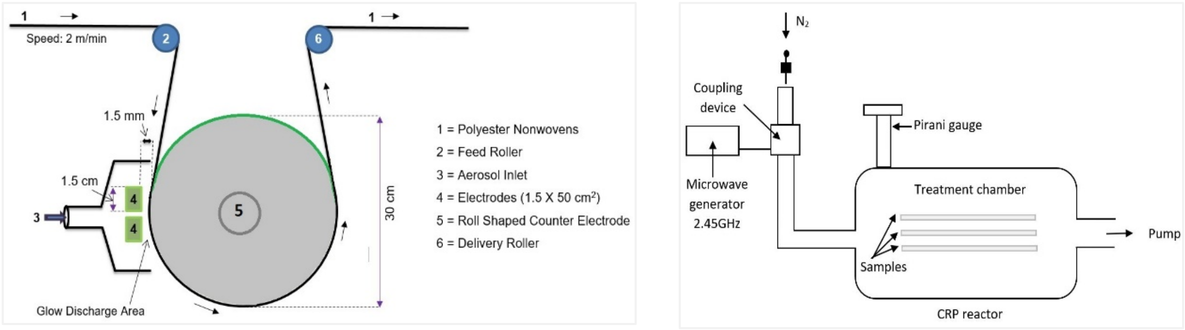

Figure S2: Schematic illustration of (a) coating star atmospheric plasma treatment and (b) cold remote plasma treatment machines

1.3. *Glucose Oxidase* activity Assay principle

Glucose oxidase activity was measured according to the enzyme's ability to catalyze the oxidation reaction of β-D-glucose. Hydrogen peroxide is released as a by-product of this reaction as shown in reaction (1). The produced H_2O_2 enters in an another reaction with p-hydroxybenzoic acid and 4-aminoantipyrine in presence of peroxidase enzyme (POD), which results in a pink colored complex (quinoneimine dye complex) at 30° C and pH 7.

$$\beta - \mathrm{D} - \mathrm{glucose} + O_2 + H_2\mathrm{O} \xrightarrow{GOx} D - glucono - \delta - lactone + H_2O_2 \quad (1)$$

$$2H_2O_2 + p - hydroxybenzoic\ acid + 4 - aminoantipyrine \xrightarrow{POD} Quinoneimine\ dye + 4H_2\mathrm{O} \quad (2)$$

The absorbance intensity of the colored complex was monitored at λ_{510nm} using a UV–vis spectrophotometer exactly after 20 min. From the absorbance difference (A_2-A_1) between blank and GOx sample $\Delta A_{510\,nm}$/20 min can be calculated. The activity values (U/L) are obtained by following calculations.

$$\mathrm{U/L\ of\ sample\ solution} = \frac{\mathrm{mU/0.5\ mL\ x\ 2000x\ D}}{1000} \quad (3)$$

Here, 2000 is the conversion from 0.5 mL as assayed to 1 L, 1/1000 is conversion from mU to U and D is dilution factor. On the other hand, in case of solid or semi solid sample, the activity (U/g) is calculated from the amount weighed as follows;

$$\mathrm{lucose\quad oxidase\ activity\ (U/g\ of\ preparation)} = \frac{\mathrm{GOx\ activity\ [U/L\ sample\ solution]}}{\mathrm{weight\ [g/L\ sample\ solution]}} \quad (4)$$

2. RESULTS AND DISCUSSIONS

2.1. Water contact angle and capillary uptake measurement

(a) Untreated polyester fabric **Θ_{H_2O} = 141°**

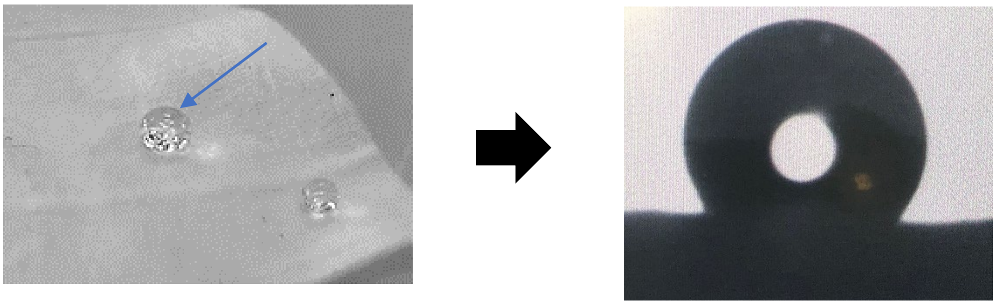

(b) Plasma treated polyester fabric **Θ_{H_2O} = 0°**

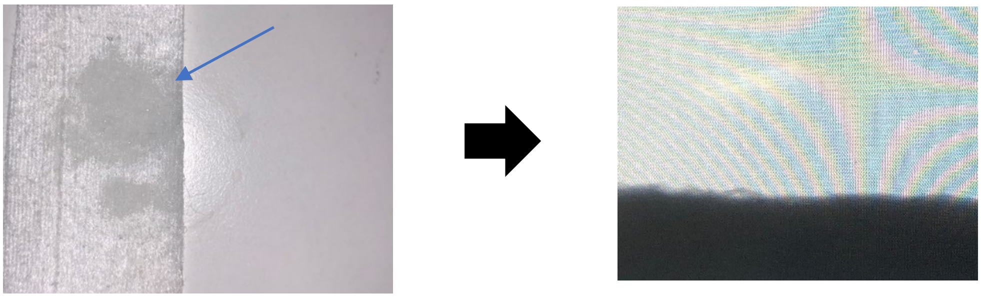

Figure S3: Water contact angle analysis of untreated (a) and plasma treated (b) polyester fabric using sessile water droplet.

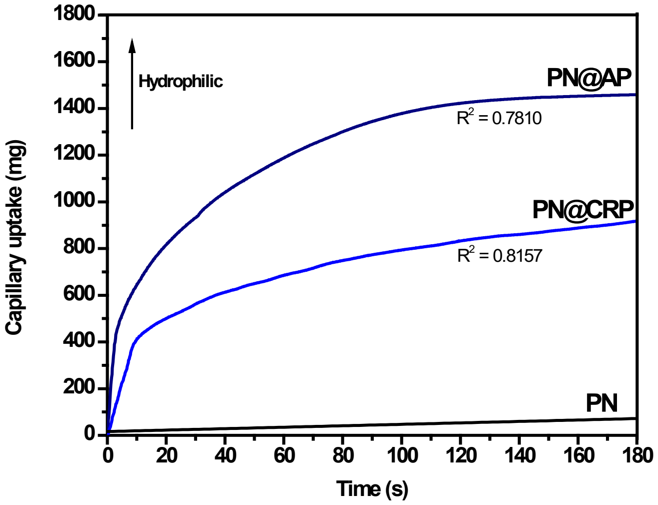

Figure S4: Relative capillary uptake of untreated, AP plasma treated and CR plasma treated polyester fabric.

2.2. X-ray Photoelectron Spectroscopy (XPS)

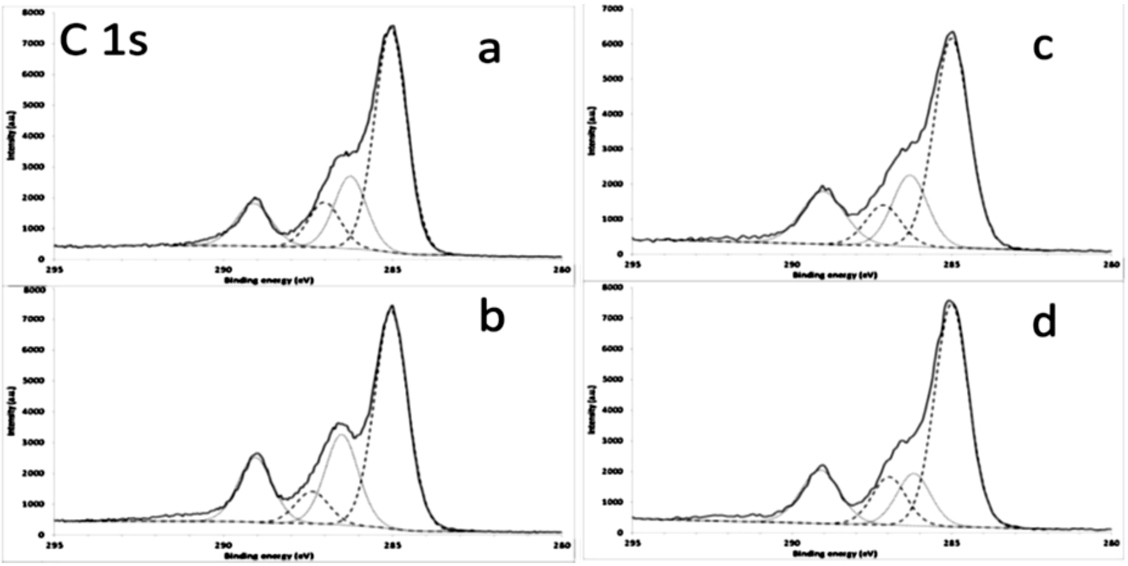

Figure S5. XPS C1s spectra of (a) untreated, (b) atmospheric pressure plasma and (c, d) cold remote plasma treated polyester surface

2.3. FTIR spectra of untreated and plasma treated polyester fabric

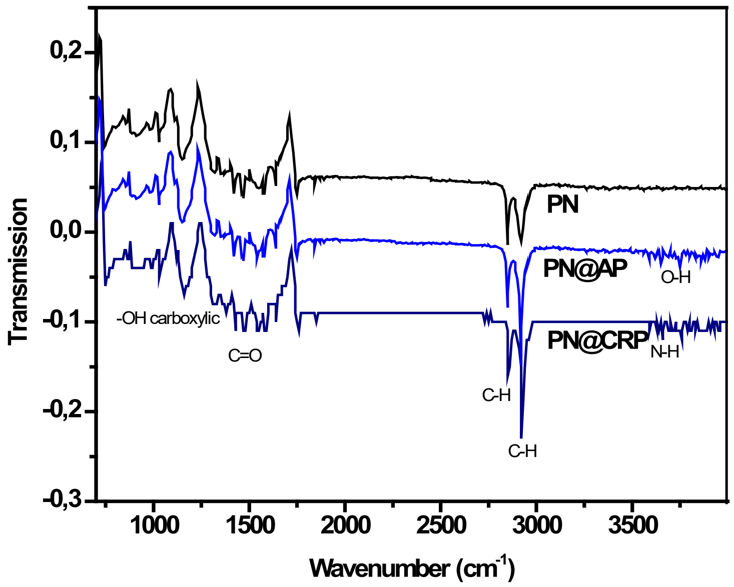

Figure S6: Fourier transform infrared spectroscopy (FTIR) spectrum of PN, PN@AP, and PN@CRP

2.4. Kinetics of GOx enzymatic reaction

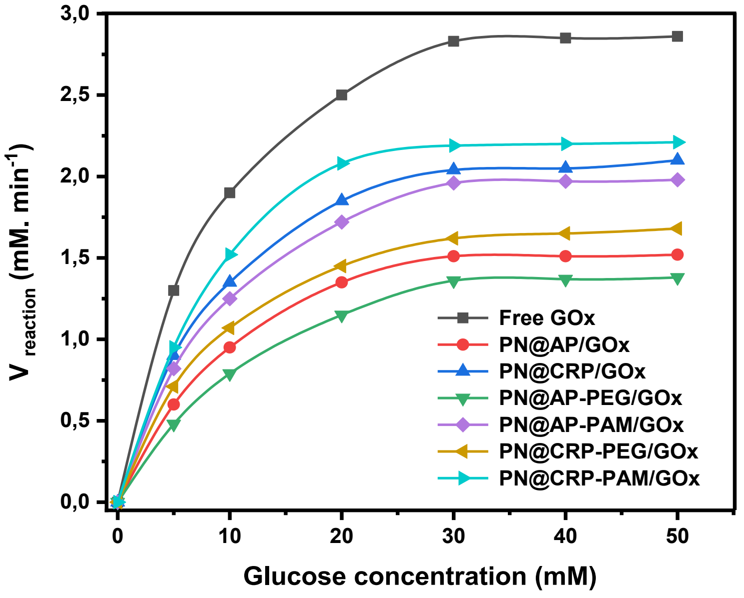

Figure S7: Reaction rates of free and immobilized GOx; Free GOx, PN@AP/GOx, PN@CRP/GOx, PN@AP-PEG/GOx, PN@AP-PAM/GOx, PN@CRP-PEG/GOx, PN@CRP-PAM/GOx.

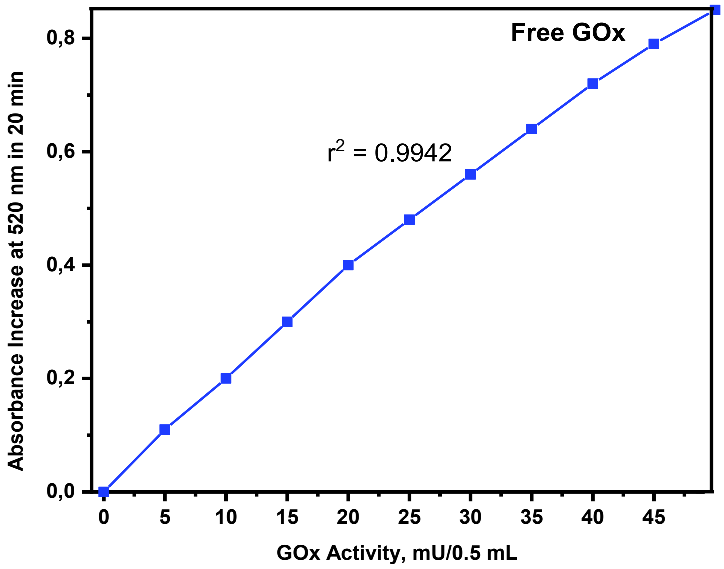

Figure S8: Standard curve relating glucose oxidase activity (mU/assay i.e. /0.5 mL) to absorbance at $\lambda_{510\ nm}$

2.5. Antibacterial activity

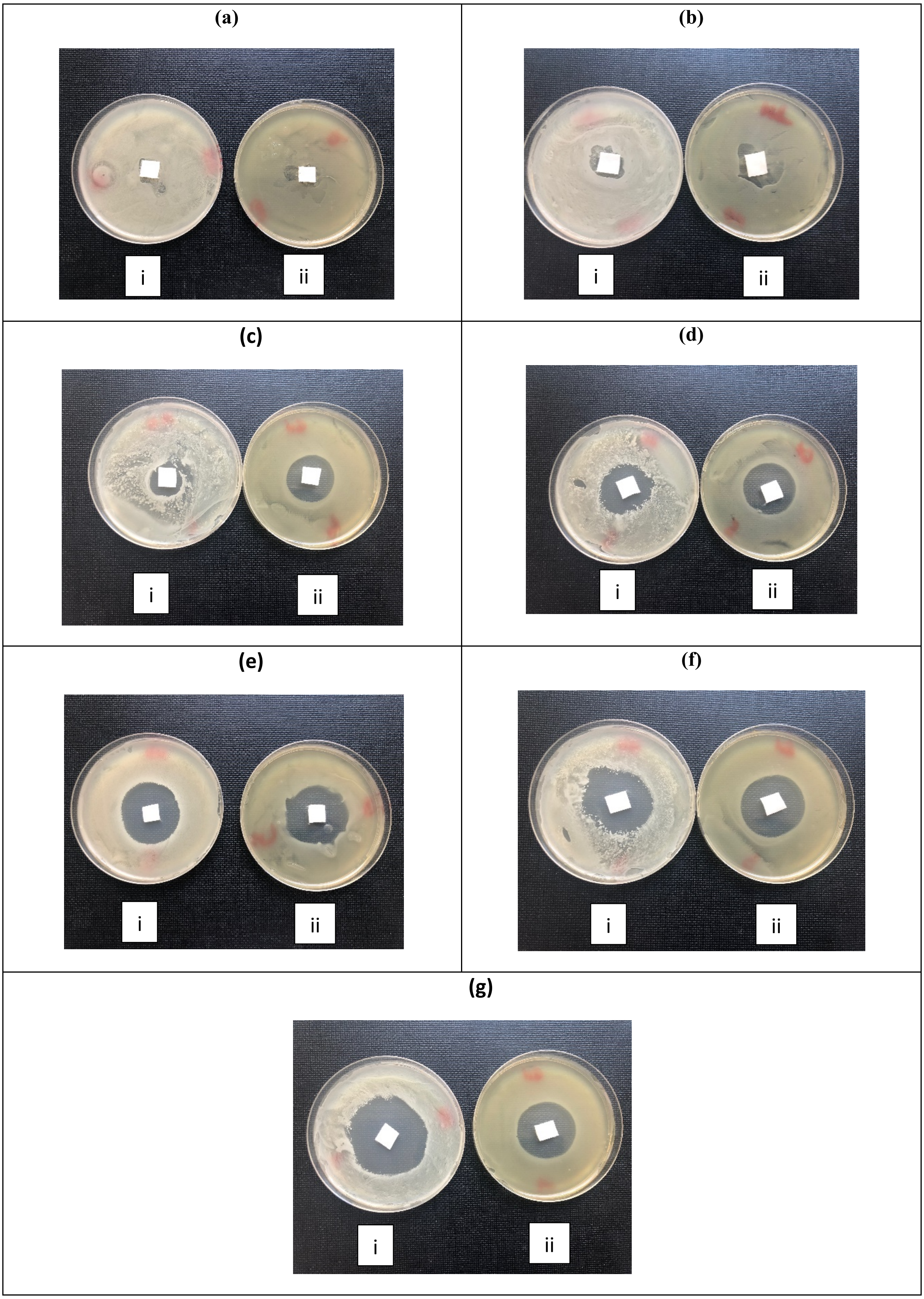

Figure S9: Zone inhibition analysis of (a) untreated PET, (b) PN@AP/GOx, (c) PN@CRP/GOx, (d) PN@AP-PEG/GOx, (e) PN@AP-PAM/GOx, (f) PN@CRP-PEG/GOx and (g) PN@CRP-PAM/GOx [(i) *Staphylococcus epidermidis* and (ii) *Escherichia coli*]

References:

1. Takke, V., et al., *Studies on the atmospheric air–plasma treatment of PET (polyethylene terephtalate) woven fabrics: effect of process parameters and of aging.* Journal of applied polymer science, 2009. **114**(1): p. 348-357.
2. Nabil, B., et al., *Development of new multifunctional filter based nonwovens for organics pollutants reduction and detoxification: High catalytic and antibacterial activities.* Chemical Engineering Journal, 2019. **356**: p. 702-716.
3. Morshed, M.N., et al., *Iron-loaded amine/thiol functionalized polyester fibers with high catalytic activities: a comparative study.* Dalton Transactions, 2019.
4. Morshed, M.N., et al., *Stabilization of zero valent iron (Fe0) on plasma/dendrimer functionalized polyester fabrics for Fenton-like removal of hazardous water pollutants.* Chemical Engineering Journal, 2019. **374**: p. 658-673.
5. Mohamed, A., et al., *Activity of enzymes immobilized on plasma treated polyester.* Journal of Molecular Catalysis B: Enzymatic, 2016. **134**: p. 261-272.
6. Tiwari, S., et al., *Influence of cold remote nitrogen oxygen plasma treatment on carbon fabric and its composites with specialty polymers.* Journal of Materials Science, 2011. **46**(4): p. 964-974.
